# The Role and Mechanism of microRNA-1224 in Human Cancer

**DOI:** 10.3389/fonc.2022.858892

**Published:** 2022-04-14

**Authors:** Mingwei Ma, Jie Li, Zimu Zhang, Juan Sun, Zhen Liu, Ziyang Zeng, Siwen Ouyang, Weiming Kang

**Affiliations:** ^1^ Chinese Academy of Medical Sciences and Peking Union Medical College, Beijing, China; ^2^ Department of General Surgery, Peking Union Medical College Hospital, Beijing, China

**Keywords:** miR-1224, cancer, tumor suppressor, function, clinical implication

## Abstract

microRNAs (miRNAs) are a type of small endogenous non-coding RNAs composed of 20-22 nucleotides, which can regulate the expression of a gene by targeting 3’ untranslated region (3’-UTR) of mRNA. Many studies have reported that miRNAs are involved in the occurrence and progression of human diseases, including malignant tumors. miR-1224 plays significant roles in different tumors, including tumor proliferation, metastasis, invasion, angiogenesis, biological metabolism, and drug resistance. Mostly, it serves as a tumor suppressor. With accumulating proofs of miR-1224, it can act as a potential bio-indicator in the diagnosis and prognosis of patients with cancer. In this article, we review the characteristics and research progress of miR-1224 and emphasize the regulation and function of miR-1224 in different cancer. Furthermore, we conclude the clinical implications of miR-1224. This review may provide new horizons for deeply understanding the role of miR-1224 as biomarkers and therapeutic targets in human cancer.

## Introduction

microRNAs (miRNAs) are endogenous non-coding small RNAs that are composed of 20-22 nucleotides and are widely present in eukaryotic cells (1). miRNAs regulate the cellular protein expression through binding to the 3’ untranslated region (3’-UTR) of the targeted mRNA, resulting in decreased or degraded expression of the target genes (2). Complete or incomplete binding of 6-8 nucleotide seed sequences of each miRNA can bind up to 100 mRNAs, leading to degradation or translation inhibition, respectively. Therefore, each miRNA can bind and regulate multiple mRNAs, and one mRNA can also be regulated by diverse miRNAs.

miRNAs are generated by endogenous transcribed primary transcripts, which are further cleaved by Drosha (RNase III) in the nucleus to produce stem-loop precursors miRNAs (pre-miRNAs) of approximately 70 nucleotides (3). Pre-miRNAs are transported by Exportin 5 from the nucleus to the cytoplasm and further processed by Dicer (RNase III) for the production of mature miRNAs (4, 5).

To date, more than 1600 miRNAs have been found and identified, most of which are highly conserved in mammalian species. The functions of miRNAs have been validated in developmental timing, cell proliferation, cell differentiation, cell apoptosis, and tumorigenesis additionally (6–10). Considerable research revealed that miRNAs are dysregulated in different tumor types, which act as tumor inhibitors or tumor promotors and actively participate in the oncogenic process (11). In addition, miRNAs also play critical roles in predicting tumor classification, treatment response, and prognosis of patients (12).

miR-1224, located at chromosome 3q27.1, is a class of mammalian mirtron encompassed in the last intron of the VWA5B2 gene (von Willebrand factor A domain containing 5B2) and is discovered that acts vital roles in some diseases, such as acute liver failure, Parkinson’s disease, and cerebral ischemia (13–15). miR-1224 has two mature sequences, miR-1224-5p and miR-1224-3p, which perform different functions, respectively. Although some research has reported that miR-1224 expressed abnormally in several tumors, its biological function and specific mechanism in different cancers are still inconsistent (16–18). Moreover, the expression profile and its potential clinical significance of miR-1224 have not been investigated. Therefore, we systematically review the role and the detailed mechanism of miR-1224 in cancer, to gain a better comprehension of its potential role as biomarkers and therapeutic targets in cancer.

## miR-1224 Expression in Human Cancer

miR-1224 was expressed variously and mostly downregulated in human cancers (**Table 1**). The expression of miR-1224 in different kinds of tumors was showed followingly.

**Table 1:** Expression profiles of miR-1224 in human cancers.

Systems	RNAs	Cancer type	Role	Expression	Sources	References
Respiratory system	miR-1224	LC	tumor suppressor	downregulation	tissue and cell	(19)
	miR-1224-5p	LP	tumor suppressor	downregulation	tissue and cell	(20)
	miR-1224-3p	LAUD	tumor suppressor	downregulation	tissue and cell	(21)
Nerve system	miR-1224-3p	LGG	tumor suppressor	downregulation	tissue	(22)
	miR-1224-3p	Glioma	tumor suppressor	downregulation	cell	(23)
	miR-1224-3p	Glioma	tumor suppressor	downregulation	tissue and cell	(24)
	miR-1224-5p	Glioma	tumor suppressor	downregulation	tissue and cell	(18)
	miR-1224-5p	GBM	tumor suppressor	downregulation	tissue and cell	(25)
	miR-1224-5p	GBM	tumor suppressor	downregulation	GEO database	(26)
Muscular and skeletal systems	miR-1224-5p	OS	tumor suppressor	downregulation	tissue and cell	(27)
	miR-1224-5p	OS	tumor suppressor	downregulation	tissue and cell	(28)
Genitourinary system	miR-1224-5p	BCa	tumor suppressor	downregulation	tissue and cell	(29)
	miR-1224-3p	BCa	tumor suppressor	downregulation	tissue	(30)
	miR-1224-5p	BCa	tumor suppressor	upregulation	tissue and cell	(31)
	miR-1224-3p	BC	tumor promotor	upregulation	cell	(32)
Digestive system	miR-1224-5p	TSCC	tumor suppressor	downregulation	cell	(33)
	miR-1224-5p	OSCC	tumor suppressor	downregulation	tissue and cell	(34)
	miR-1224	GC	tumor suppressor	downregulation	tissue and cell	(35)
	miR-1224-5p	GC	tumor suppressor	downregulation	tissue and cell	(36)
	miR-1224	intestinal-type GC	tumor suppressor	downregulation	tissue and cell	(37)
	miR-1224-5p	CRC	tumor suppressor	downregulation	tissue and cell	(38)
	miR-1224-5p	CRC	tumor suppressor	downregulation	tissue and cell	(39)
	miR-1224-5p	CRC	tumor suppressor	downregulation	tissue	(17)
	miR-1224-5p	ESCC	tumor suppressor	downregulation	tissue and cell	(40)
	miR-1224-5p	ESCA	tumor suppressor	downregulation	tissue and cell	(41)
	miR-1224	HCC	tumor suppressor	downregulation	tissue and cell	(42)
	miR-1224	HCC	tumor suppressor	downregulation	tissue and cell	(43)
	miR-1224-5p	HCC	tumor suppressor	downregulation	cell	(44)
	miR-1224-5p	PC	tumor suppressor	downregulation	tissue and cell	(45)
	miR-1224-5p	PC	tumor suppressor	downregulation	tissue and cell	(46)
Skin	miR-1224-5p	Melanoma	tumor suppressor	downregulation	tissue and cell	(47)
	miR-1224-5p	keloids	tumor suppressor	downregulation	tissue and cell	(48)

In the respiratory system, especially in lung cancer, miR-1224 was usually downregulated. miR-1224 was lower level detected by quantitative reverse transcription PCR (qRT-PCR) in lung cancer tissues than normal lung tissues (19). Zuo et al. found that miR-1224-3p was decreased in lung adenocarcinoma (LAUD) tissues compared to that in normal tissues *via* qRT-PCR method. Transcriptional profiling studies also showed that miR-1224-3p was remarkably reduced in LAUD cell lines (21). In the tissues of laryngeal papillomas (LP), further research proved that miR-1224-5p was greatly decreased by using qPCR. In addition, miR-1224-5p was downregulated in LP cell lines when compared to normal cells (20).

In the nervous system, miR-1224 was mostly downregulated in nervous system neoplasms (18, 23, 24). miR-1224-3p was reduced in glioma by using miRNA assay and real time PCR (23, 24), which was also observed in low-grade glioma (LGG) from GEO and TCGA database (22). Qian et al. also confirmed that miR-1224-5p was downregulated in glioma by *in situ* hybridization of tissue samples (18). In glioblastoma (GBM), Xu et al. and Xiong et al. reported that miR-1224-5p acted as a tumor suppressor and a significant reduction of miR-1224-5p was detected in GBM tissues and cell lines *via* qRT-PCR and GEO database, respectively (25, 26).

Reduced miR-1224 was common in the muscular and skeletal system. For instance, miR-1224-5p was decreased in osteosarcoma (OS) tissues and cell lines by qRT-PCR (27, 28).

Similarly, miR-1224 was reduced in the digestive system. miR-1224-5p was downregulated in the cells of tongue squamous cell carcinoma (TSCC) and oral squamous cell carcinoma (OSCC) by RT-qPCR analysis (33, 34). In gastric cancer (GC), miR-1224 was reduced in GC tissues and cell lines through RT-PCR analysis (35–37). In colorectal cancer (CRC), miR-1224-5p also acted as a tumor suppressor and a significant reduction of miR-1224-5p was detected in CRC tissues and cell lines according to qRT-PCR, western blot, and immunohistochemistry (17, 38, 39). In hepatocellular carcinoma (HCC) and pancreatic cancer (PC), miR-1224 showed a descending trend, especially the miR-1224-5p according to bioinformatics analysis (GEO datasets), RNA sequencing and qRT-PCR validation (42–46). Not only that, but miR-1224-5p also declined in esophageal squamous cell carcinoma (ESCC) and esophageal cancer (ESCA) tissues compared to normal tissues by qRT-PCR (40, 41).

miR-1224 was somewhat controversial in the genitourinary system. Through qRT-PCR experiments, miR-1224 was found downregulated in bladder cancer (BCa) (29, 30). However, Ding et al. reported that miR-1224-5p was elevated in the BCa tissues and cell lines using TCGA database (31). Similarly, Ran et al. found that miR-1224-3p was increased in breast cancer (BC) cells by using RT-qPCR methods (32).

In skin system, miR-1224-5p was significantly downregulated in melanoma tissues and cell lines by using qRT-PCR (47). miR-1224-5p was also downregulated in keloids from miRNA microarray and qRT-PCR (48).

Those data proved that there was wide diversity for miR-1224 expression in different cancers, sometimes even in same cancer.

## The Regulation of miR-1224 in Human Cancer

Generated by non-coding mRNA splicing, miR-1224 was regulated by multiple signaling molecules such as CREB1, SND1, and β-catenin (**Table 2**). LncRNAs mainly performed as ceRNA to sponge miRNAs and thus to regulate miRNA expression. In LC, miR-1224 was repressed by long-chain non-coding RNA (lncRNA) NEAT1, thereby upregulating KLF3 expression (19). LncRNA NEAT1 also regulated miR-1224-5p in GC by sponging miR-1224-5p, thus regulating RSF1 expression and in turn, altering the evolution of GC (36). Additionally, Linc00460 regulated miR-1224-5p in OS and ESCA, respectively (28, 41). Linc00460 functioned as a molecular sponge to absorb miR-1224-5p, thereby promoting metastasis and epithelial-to-mesenchymal transition (EMT) of ESCA and OS progression (28, 41). There were other lncRNAs to regulate miR-1224 besides LncRNA NEAT1 and Linc00460. Linc00665 was the sponge for miR-1224-5p, which elevated the SND1 in PC cells (46). Zhao et al. discovered that LncRNA IGFL2-AS1 played an oncogenic role in TSCC (33). It interacted with miR-1224-5p to regulate SATB1, which activated the transcriptional activity of Wnt/β‐catenin in TSCC cells (33). LncRNA ZEB1-AS1 was generated from the promoters of ZEB1, which played a vital role in tumorigenesis. Experimental data indicated that ZEB1-AS1 directly regulated miR-1224-5p, thus controlling the processes of development and progression in melanoma (47). To sum up, several articles have confirmed that multiple lncRNA molecules are involved in the regulation of miRNA, mainly acting as sponges to inhibit miRNA expression.

**Table 2:** Upstream regulations and biological functions of miR-1224 involved in different cancers.

Systems	Cancer type	RNAs	Upstream gene	Biological functions	References
Respiratory system	LC	miR-1224	NEAT1	Inhibit proliferation and invasion, promote apoptosis	(19)
	LAUD	miR-1224-3p	Circ-ZNF609	Inhibit proliferation and cell cycle	(21)
Nerve system	Glioma	miR-1224-3p	Circ-ZNF609	Inhibit proliferation, migration and invasion	(24)
		miR-1224-3p	EZH2	Inhibit proliferation, invasion and glucose metabolism	(23)
	GBM	miR-1224-5p	MIR44435‐2HG	Inhibit proliferation and invasion, promote apoptosis	(25)
Muscular and skeletal systems	OS	miR-1224-5p	linc00460	Inhibit proliferation, invasion and migration	(28)
		miR-1224-5p	\	Inhibit proliferation, invasion and EMT, promote apoptosis, autophagy	(27)
Genitourinary system	Bca	miR-1224-5p	circCASC15	Inhibit proliferation	(29)
		miR-1224-5p	FOXI1	Inhibit viability, migration and invasion	(31)
	RCC	miR-1224-3p	circ-EGNL3	Inhibit proliferation, invasion, and migration	(49)
	BC	miR-1224-3p	\	Inhibit apoptosis, promote EMT, migration and metastasis	(50)
		miR-1224-3p	\	Promote cell growth and metastasis	(32)
Digestive system	TSCC	miR-1224-5p	IGFL2-AS1	Inhibit proliferation, migration, invasion and EMT	(33)
	OSCC	miR-1224-5p	APCDD1L-AS1	Inhibit proliferation and promote apoptosis	(34)
	GC	miR-1224-5p	NEAT1	Inhibit proliferation, invasion, and migration	(36)
		miR-1224	\	Inhibit proliferation, migration, invasion, and EMT	(35)
	HCC	miR-1224	CREB	Inhibit proliferation and cell cycle	(42)
		miR-1224	circRASGRF2	Inhibit proliferation, cell cycle, invasion, migration and EMT, promote apoptosis, autophagy	(43)
		miR-1224-5p	/	Inhibit proliferation, migration and invasion, promote apoptosis	(44)
	CRC	miR-1224-5p	Circ-RNF121	Inhibit proliferation, migration, invasion and glycolysis, promote apoptosis	(38)
		miR-1224-5p	/	Inhibit migration, invasion and EMT	(39)
	ESCA	miR-1224-5p	Linc00460	Inhibit migration, invasion and EMT	(41)
	PC	miR-1224-5p	Linc00665	Inhibit proliferation, migration and invasion	(46)
		miR-1224-5p	/	Inhibit proliferation, migration, invasion and EMT	(45)
Skin	Melanoma	miR-1224-5p	ZEB1-AS1	Inhibit proliferation, migration and invasion	(47)
	Keloids	miR-1224-5p	/	Inhibit proliferation, migration and invasion, promote apoptosis	(48)

Circular RNAs (circRNAs) are identified as a type of endogenous non-coding RNAs and exist conserved miRNA target sites, and therefore circRNAs could act as miRNA sponges to modulate its expression. Circ-CASC15 was highly expressed in BCa, which directly bind to miR-1224-5p. Consequently, CREB1, the target of miR-1224-5p, was increased in BCa (29). Recent studies have shown that circ-EGLN3 was involved in RCC tumorigenesis through downregulating miR-1224-3p, which targeted HMGXB3, thus regulating proliferation, invasion, and migration (49). CircRNAs also played an important role in the progression of digestive system neoplasms, such as circ-RASGRF2 and circ-RNF121 (38, 43). Circ-RASGRF2 was originated from RASGRF2 and identified to be remarkably upregulated in HCC. Further data confirmed that circRASGRF2 facilitated the expression of FAK by sponging miR-1224. The knockdown of circ-RASGRF2 inhibited the proliferation and migration of HCC cells. (43). Circ-RNF121 was remarkably upregulated in CRC. Similarly, circ-RNF121 functioned as a sponge of miR-1224-5p to regulate cell growth, migration, and invasion in CRC (38). Zuo et al. found that circ-ZNF609 sponged miR-1224-3p to downregulate its expression in LAUD. As a result, the molecular target of miR-1224-3p, ETV1, was upregulated in LAUD. Recently, ETV1 has been identified to play an oncogenic role (21). In nerve system, Circ-ZNF609 functioned as a miR-1224-3p sponge and mediated cell behaviors in glioma. It promoted cell proliferation and metastasis by promoting PLK1 *via* binding to miR-1224-3p competitively (24). Other regulatory factors modulated miR-1224-3p expression in gliomas, such as EZH2 and MIR44435‐2HG (lncRNA MIR4435‐2 Host Gene) (23, 25). EZH2, a core component of PRC2, acted as a histone methyltransferase that trimethylated histone 3 at lysine 27 (H3K27me3), silencing the gene. In gliomas, miR-1224-3p was inhibited by EZH2, which in turn regulated β-catenin expression through binding to its 3′UTR, thus controlling proliferation, invasion, and glucose metabolism of cells (23). MIR44435‐2HG belonged to long non‐coding RNAs and was involved in the regulation of brain tumor progression. Knockdown of MIR4435‐2HG contributed to the inhibition of cell proliferation and invasion of GBM. MIR4435‐2HG suppressed miR-1224-5p expression through similar mechanism (25).

## The Function of miR-1224 in Human Cancer

**Figure 1:**
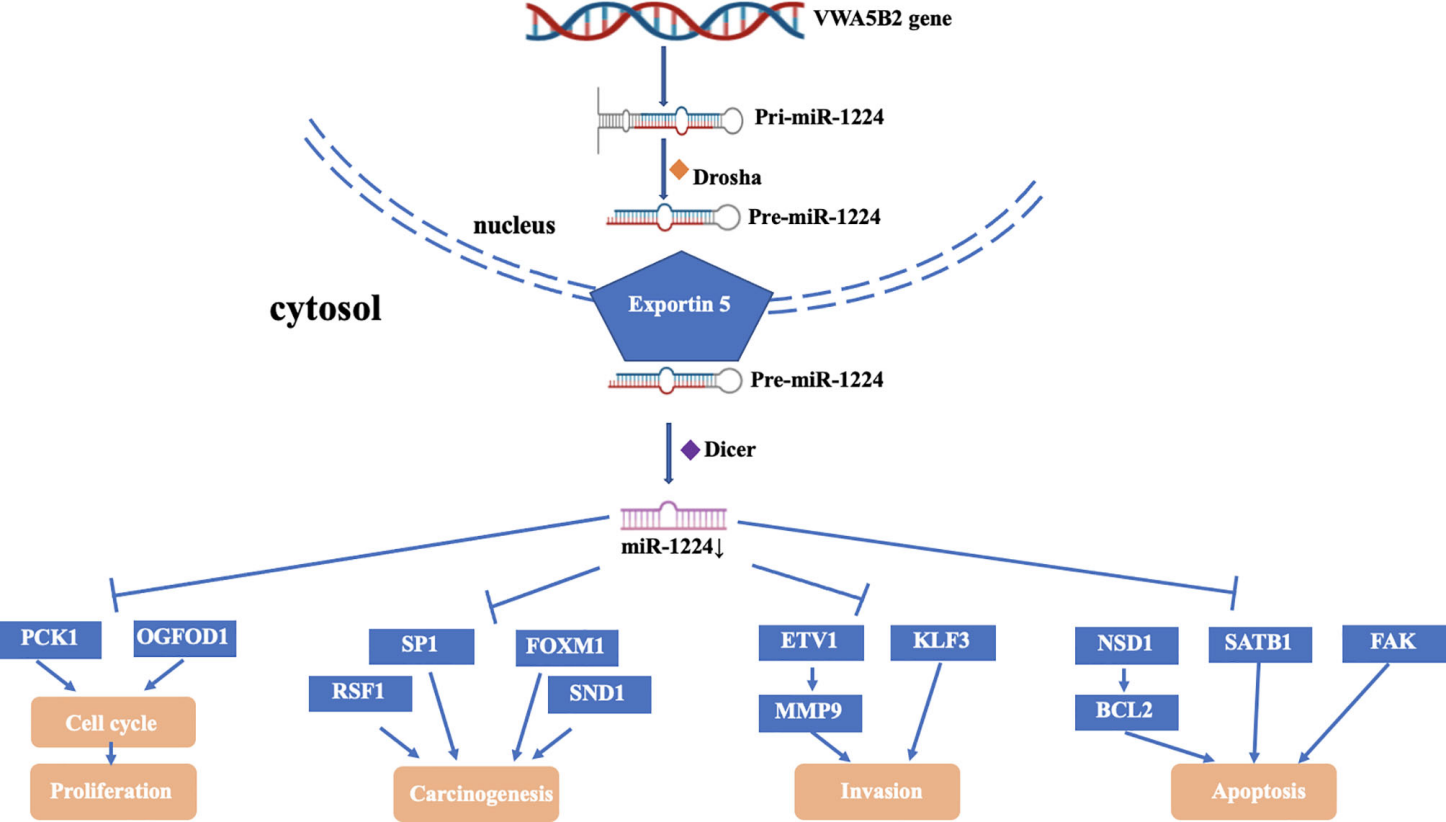
Mechanism and function of miR-1224 downregulation in cancer.

As a tumor suppressor, miR-1224 significantly inhibited the proliferation, migration, and invasion and induced apoptosis of cancer cells (33). Additionally, miR-1224 participated in the process of the cell cycle, apoptosis, autophagy, and EMT to repress development of tumor (40). Also, miR-1224 influenced metabolic behavior such as glucose metabolism to inhibit the cell growth of cancer (1). Interestingly, miR-1224 promoted the migratory ability of cells and induced EMT in BCa and triple-negative breast cancer (TNBC) (32, 50). In the following part, we systematically proposed the functions of miR-1224, including oncogenic factors and tumor suppressors (**Figure 1**).

## Inhibition of the Cancerogenic Process

Proto-oncogenes normally promoted cell division and proliferation, playing a vital role in the early stages of growth and development. When proto-oncogenes mutated, such as point mutation, gene amplification, chromosomal translocation, promoter insertion, the proto-oncogenes were over-activated and transformed to oncogenes, resulting in excessive cell growth, eventually leading to the initiation and progression of tumors. Abundant studies have found that FOXM1, as an oncogene, was generally highly expressed in tumors. Furthermore, it was implicated in all key features of the cancers described by Hanahan and Weinberg. FOXM1 induced oncogenic WNT and TGFβ signaling pathways by interacting with other proteins such as β-catenin or SMAD3 (51). Jiang et al. revealed that miR-1224 can bind to FOXM1 in CRC cells and inhibited its function, thus blocking the occurrence of cancer (38). Similarly, the oncogenic effects of other oncogenes, such as SP1, RSF1, and SND1, were attenuated when miR-1224 was co-present with them (36, 39, 52).

## Promotion of Cell Apoptosis and Autophagy

Currently, many anti-cancer therapies were targeting molecules involved in cell apoptosis regulation (53–55). BCL2 and BAD belonged to the BCL2 family, which controlled the internal apoptosis pathway. On the whole, BCL2 played a part in anti-apoptotic, while BAD played a part in pro-apoptotic. Recent studies have indicated that NSD2 deficiency repressed the expression of BCL2 but upregulated the expression of BAD (55). However, NSD2 appeared to play an antiapoptotic role in OSCC cells, and its elevated expression was associated with the poor prognosis of OSCC patients. However, miR-1224 reversed the antiapoptotic effects of NSD2 and promoted cell apoptosis by binding to NSD2 (34). SATB1 and FAK played an active function in the apoptotic cleavage of cellular proteins, similarly, miR-1224 accelerated the cell apoptosis *via* targeting SATB1 and FAK in cancer (33, 43). Autophagy was a conserved catabolic biological process widely existing in eukaryotes and lysosomes that participated in digestion and degradation of their macromolecules or damaged organelles to finish their biological functions (56). Autophagy was a double-edged sword in tumor progression (57). It can not only inhibit the formation of tumors but also assist cells to fight against hypoxic condition, lack of nutritional factors, and other adverse growth environments, thus boosting the initiation and progression of tumors. Zhao et al. discovered that FADS1 regulated the process of autophagy in laryngeal squamous cell carcinoma through activating AKT/mTOR signaling (58). A recent study found that miR-1224 restrained the expression of FADS1 in OS (28). Therefore, miR-1224 played a role in promoting autophagy through binding to different targets. Not only that, but miR-1224-5p also inhibited OS autophagy by targeting the PLK1-mediated PI3K/AKT/mTOR pathway. It was well known that autophagy-related molecules such as LC3-II/I, P62, and Beclin-1 can regulate autophagy activity during the autophagy process. Jin et al. have found that miR-1224-5p significantly facilitated the expression of LC3II/I and Beclin-1 which were autophagy-related in OS by targeting PLK1 (27).

## Suppression of Cell Invasion

The invasion of a malignant tumor referred to the invasion and diffusion of cells to the surrounding environment. The direct diffusion of cells to the surrounding area without separating from the main body of the tumor was called direct diffusion without metastasis. Cells invaded blood vessels, lymphatics, and body cavities, then were removed from the main body of the tumor and continued to grow in distant organs, forming new tumors of the same type, which was called metastasis. The highly invasive characteristics of tumors were associated with a poor prognosis. Upregulated MMPs were involved in cell migration and invasion (59). Oh et al. disclosed that MMPs were regulated by ETVs, and emphasized that ETV1 was the most important one (60). Recent studies demonstrated that miR-1224 bound to the 3’-UTR of ETV1 to reduce its expression, and overexpressed miR-1224 suppressed ETV1 and MMPs, which significantly inhibited the invasion of cells (21). miR-1224 blocked the translation of KLF3 by binding to the mRNA, and inhibition of miR-1224 led to an increase of KLF3, thus enhancing the aggressiveness of cells (19). These results suggested that miR-1224 played a critical role in the regulation of cell migration and invasion through directly interacting with ETV1 and regulating MMPs, known targets of ETV1.

## Induction of Cell Cycle Arrest

Mitosis is one of the most important steps in the cell cycle (57). Du et al. reported that miR-1224 was frequently downregulated in glioma, the miR-1224-3p inhibitor significantly reduced the expression of miR-1224-3p and remarkably accelerated the cell proliferation. Another study discovered that miR-1224-3p was bound to PLK1, which was involved in mitosis and the cell cycle. The abovementioned data revealed that miR-1224-3p inhibited tumor growth by directly binding to PLK1 (24). OGFPD1, a stress granule protein, was linked closely to cell cycle G1/2 and G1/M. Recent studies found a significantly increased expression of OGFOD1 in LP tissues and cells, which was associated with the promotion of cell viability and proliferation in LP. Overexpressed miR-1224-5p significantly inhibited OGFOD1-induced cell proliferation and activity by targeted OGFOD1 (20).

## Role in EMT

Multiple studies showed that reduced miR-1224 enhanced the invasion and metastasis of a variety of tumors. Oh et al. discovered that miR-1224 was downregulated in the process of EMT (60). In addition, miR-1224 indirectly affected differentiation and EMT by inhibiting metastasis through a network of pre-metastasis stimulators that targeted VEGF, COX2, and MMP9, which were involved in angiogenesis, collagen remodeling, and proteolysis (21).

## Tumor-oncogenic Role in Some Tumors

**Figure 2:**
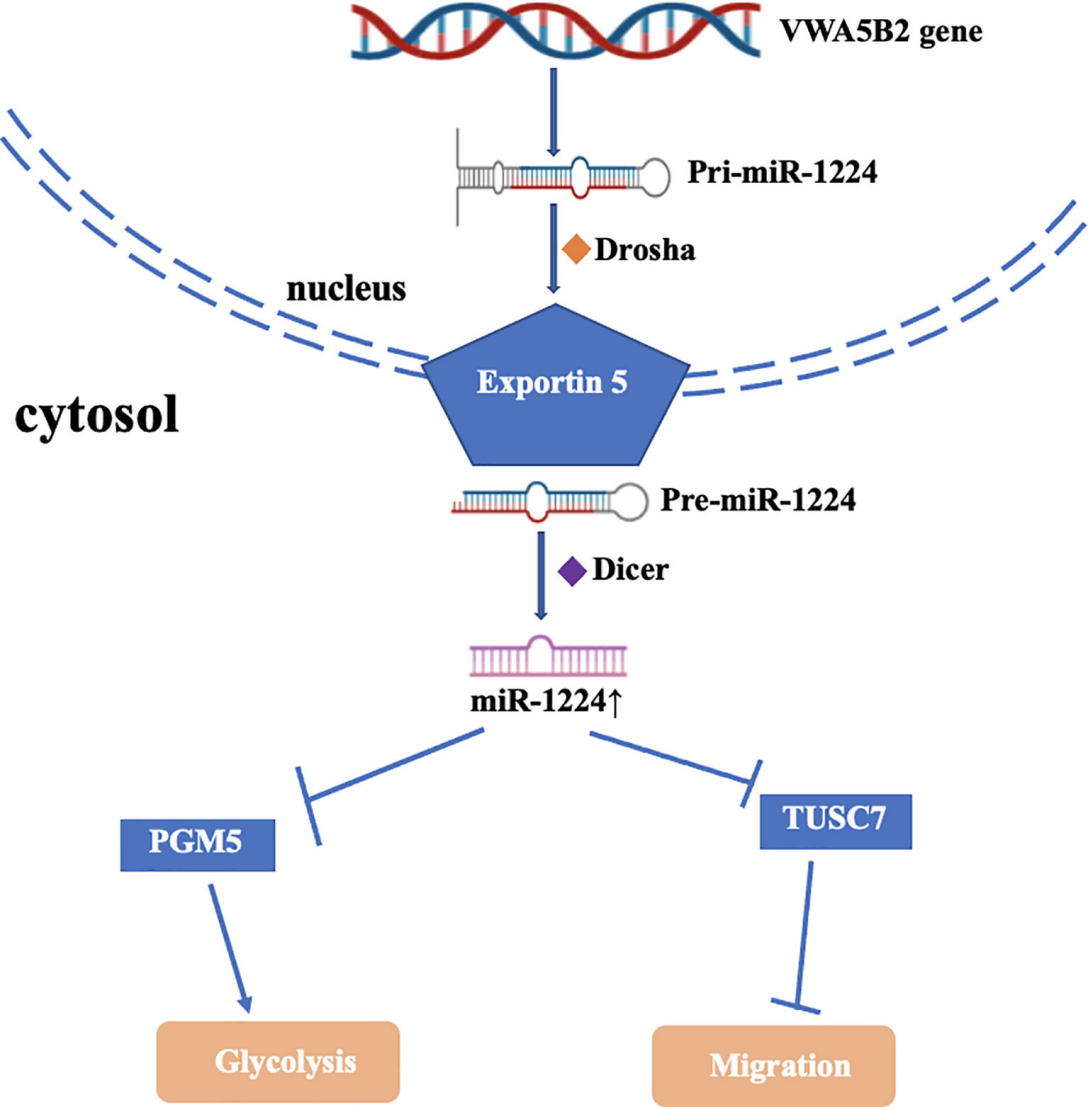
Mechanism and function of miR-1224 upregulation in cancer.

miR-1224 not only acted as a tumor suppressor but sometimes as an oncogene that promoted tumor genesis and development (**Figure 2**). As we all known, one of the signature features of cancer cells was metabolic reprogramming of aerobic glycolysis. PGM5 (A member of the phosphoglucomutase (PGM) group superfamily) catalyzed the bidirectional interconversion metabolism of glucose-1-phosphate (G1P) and glucose-6-phosphate (G6P). A recent study found that miR-1224-3p promoted cell proliferation and migration *via* PGM5-mediated aerobic glycolysis in BC (32). Another study revealed that the high expression of miR-1224-3p was an independent prognostic indicator of poor overall survival of TNBC patients. miR-1224-3p bound to TUSC7, which inhibited cell growth, proliferation, and metastasis both *in vitro* and *in vivo* in BC (50).

## Clinical Implication

The occurrence of the tumor was caused by many factors, and its progression was directly related to the therapeutic effects of patients (61). Therefore, early diagnosis and individual treatment were critical for the patients to prolong their survival. Tumor markers were widely used in the screening of many tumors, but the low sensitivity of tumor markers made the results inaccurate and conflicting (62). The differential expression of miRNAs in tumor tissues became the focus of research, which can be combined with the detection of tumor markers to facilitate the screening and prognosis of tumors.

Many research indicated that miR-1224 can be used as a prognostic biomarker in clinical practice (**Table 3**). When miR-1224 acted as tumor-suppressors, reduced miR-1224 indicated a short overall survival for patients with malignant tumors. Zhao et al. compared the expression of miR-1224-5p in TSCC cells with that in normal cells and found that miR-1224-5p was decreased in TSCC cells, suggesting miR-1224 may aid as a new biomarker contributing to TSCC treatments (33). Patients who developed with III+IV stage had a higher miR-1224 expression, suggesting that it can act as a brand-new biomarker for GC patients (35). Wang et al. reported that miR-1224-5p was negatively correlated with lymph node metastasis and FIGO stage in OC, indicating miR-1224-5p was associated with survival of patients with OC (52). Shi et al. found that decreased miR-1224-5p was associated with a high TNM stage thus are an unfavorable prognostic factor for ESCC patients (40). Patients with decreased miR-1224-5p had a poor survival probability (P=0.006) in PC (45). Most Studies demonstrated that miR-1224 served as a blood-based biological indicator for early diagnosis and potential prognostic biomarker in BCa, melanoma, keloid (29, 47, 48). In contrast, Zheng et al. found that high miR-1224-3p expression was an independent clinical blood-based factor of poor OS for TNBS patients (50).

**Table 3:** Clinical implication of miR-1224 in human cancers.

Systems	Cancer type	Aggressive phenotype of low miR-1224	OS of low miR-1224	Therapeutic target	Drug resistance	References
Respiratory system	LC	Yes	Poor	Yes	/	(19)
	LP	/	/	Yes	/	(20)
	LAUD	/	Poor	Yes	/	(21)
Nerve system	LGG	/	Poor	Yes	/	(22)
	Glioma	High grade	Poor	Yes	/	(24)
	GBM	/	Poor	Yes	PDGF receptor resistance	(25)
Muscular and skeletal systems	OS	/	Poor	Yes	/	(27)
Genitourinary system	BCa	High TNM stage	Poor	Yes	/	(29)
	BC	/	Good	Yes	/	(32)
Digestive system	TSCC	/	Poor	/	/	(33)
	OSCC	/	Poor	/	5-FU resistance	(34)
	GC	/	Poor	Yes	/	(35)
	CRC	Yes	Poor	Yes	/	(39)
	ESCC	High grade	Poor	Yes	EGFR resistance	(40)
	HCC	High TNM stage	Poor	Yes	/	(42)
	PC	High TNM stage	Poor	Yes	/	(45)
Skin	Melanoma	High TNM stage	Poor	Yes	/	(47)
	keloids	/	Poor	Yes	/	(48)

Additionally, miR-1224 can act as therapeutic targets in cancer treatment through understanding its mechanism and function (**Table 3**). Zhang et al. demonstrated that the miR-1224-3p/HMGXB3 axis can be used as a target for the treatment of RCC (49). Yang et al. identified a miR-1224/CREB feedback loop, suggesting that blocking this circuit can be a potential molecular treatment for HCC patients (42). Similarly, miR-1224-3p/PGM5 axis played a vital role in cell proliferation, metastasis, and migration, and may be a potential target for therapy of BC (32). Li et al. demonstrated that miR-1224-5p/NSD2 axis participated in the resistance to chemotherapy of 5-FU in OSCC, providing a novel target (34). MiR-1224 can be used as a therapeutic target for CRC, GC, and LAUD in given that abundant research (17, 21, 35, 39).

## Conclusions and Prospects

At present, diverse tumors with high morbidity and mortality brought heavy burdens for patients and their families. Many studies contributed to revealing the etiology of tumor occurrence and exploring effective therapeutic methods. However, the mechanism of tumor genesis, metastasis, and drug resistance is still not clear. Researchers found that miR-1224 expression in many tumor tissues and cells was significantly different from those in normal tissues and cells. miR-1224 mostly acted as a tumor suppressor in tumor initiation and development, including proliferation, metastasis, blood formation, invasion, and drug resistance. Studies have shown that miR-1224 can be used as a tumor biomarker for early diagnosis and prognosis prediction in the future.

In conclusion, with further research on miR-1224, the mechanism of miR-1224 in the occurrence and development of tumors will be gradually revealed. miR-1224 can not only serve as an indicator of tumor diagnosis and prognosis but also become an effective target for tumor therapy, providing a new direction for targeted precision therapy.

## Author Contributions

MM, JL, and WK generated this conception. MM and JL wrote this manuscript and were co-first authors. JS and ZMZ searched and collected the relative articles. ZL, ZYZ, and SO collected the data and produced the tables and figures. WK supervised and revised the manuscript. All authors contributed to the article and approved the submitted version.

## Funding

This study was funded by the CSCO-ROCHE Research Fund (No. Y-2019 Roche-015), Beijing Xisike Clinical Oncology Research Foundation (Y-HS2019-43), Wu Jieping Medical Foundation (No. 320. 6750.19020, No. 320.6750.2020-08-32), and CAMS Innovation Fund for Medical Sciences (2020-I2M-C&T-B-027).

## Conflict of Interest

The authors declare that the research was conducted in the absence of any commercial or financial relationships that could be construed as a potential conflict of interest.

## Publisher’s Note

All claims expressed in this article are solely those of the authors and do not necessarily represent those of their affiliated organizations, or those of the publisher, the editors and the reviewers. Any product that may be evaluated in this article, or claim that may be made by its manufacturer, is not guaranteed or endorsed by the publisher.

## Abbreviation

TNBC, Triple-negative breast cancers; TSCC, Tongue squamous cell carcinoma; RCC, Renal cell carcinoma; HCC, Hepatocellular carcinoma; LGG, Low-grade glioma; BC, Breast cancer; OSCC, Oral squamous cell carcinoma; CRC, Colorectal cancer; OS, Osteosarcoma; GC, Gastric cancer; LAUD, Lung adenocarcinoma; BCa, Bladder cancer; LP, Laryngeal papilloma; GBM, Glioblastoma; OC, Ovarian cancer; RC, Rectal cancer; ESCC, Esophageal squamous cell carcinoma; PC, Pancreatic cancer; ESCA, Esophageal cancer; LC, Lung cancer; EMT, Epithelial-to-mesenchymal transition; NEAT1, Nuclear paraspeckle assembly transcript 1; CGGA, Chinese Glioma Genome Atlas.
